# The “Naked Coral” Hypothesis Revisited – Evidence for and Against Scleractinian Monophyly

**DOI:** 10.1371/journal.pone.0094774

**Published:** 2014-04-16

**Authors:** Marcelo V. Kitahara, Mei-Fang Lin, Sylvain Forêt, Gavin Huttley, David J. Miller, Chaolun Allen Chen

**Affiliations:** 1 Departamento de Ciências do Mar, Universidade Federal de São Paulo, Santos, São Paulo, Brazil; 2 Centro de Biologia Marinha (CEBIMar), Universidade de São Paulo, São Sebastião, São Paulo, Brazil; 3 School of Pharmacy and Molecular Sciences, James Cook University, Townsville, Queensland, Australia; 4 Biodiversity Research Centre, Academia Sinica, Taipei, Taiwan; 5 ARC Centre of Excellence for Coral Reef Studies, James Cook University, Townsville, Queensland, Australia; 6 Research School of Biology, Australian National University, Canberra, Australian Capital Territory, Australia; 7 John Curtin School of Medical Research, Australian National University, Canberra, Australian Capital Territory, Australia; 8 Institute of Oceanography, National Taiwan University, Taipei, Taiwan; 9 Taiwan International Graduate Program (TIGP)-Biodiversity, Academia Sinica, Taipei, Taiwan; Heriot-Watt University, United Kingdom

## Abstract

The relationship between Scleractinia and Corallimorpharia, Orders within Anthozoa distinguished by the presence of an aragonite skeleton in the former, is controversial. Although classically considered distinct groups, some phylogenetic analyses have placed the Corallimorpharia within a larger Scleractinia/Corallimorpharia clade, leading to the suggestion that the Corallimorpharia are “naked corals” that arose via skeleton loss during the Cretaceous from a Scleractinian ancestor. Scleractinian paraphyly is, however, contradicted by a number of recent phylogenetic studies based on mt nucleotide (nt) sequence data. Whereas the “naked coral” hypothesis was based on analysis of the sequences of proteins encoded by a relatively small number of mt genomes, here a much-expanded dataset was used to reinvestigate hexacorallian phylogeny. The initial observation was that, whereas analyses based on nt data support scleractinian monophyly, those based on amino acid (aa) data support the “naked coral” hypothesis, irrespective of the method and with very strong support. To better understand the bases of these contrasting results, the effects of systematic errors were examined. Compared to other hexacorallians, the mt genomes of “Robust” corals have a higher (A+T) content, codon usage is far more constrained, and the proteins that they encode have a markedly higher phenylalanine content, leading us to suggest that mt DNA repair may be impaired in this lineage. Thus the “naked coral” topology could be caused by high levels of saturation in these mitochondrial sequences, long-branch effects or model violations. The equivocal results of these extensive analyses highlight the fundamental problems of basing coral phylogeny on mitochondrial sequence data.

## Introduction

The order Scleractinia, comprising the anthozoan cnidarians that produce a continuous external aragonitic skeleton [1], are not only the architects of some of the most complex habitats (i.e. coral reefs) but are also near ubiquitous in distribution. Despite their global significance [2]–[7], several key aspects of scleractinian evolution are as yet poorly understood. Most coral families are first identifiable in the Triassic, by which time much of the extant morphological diversity is represented. Molecular data implies a deep split of extant corals between two large clades, the “Complex” and “Robust” [8]–[17], but many families defined by morphology are not monophyletic by molecular criteria and some are split between “Complex” and “Robust” clades [9]–[11], [14], [17]–[20]. One hypothesis to explain the sudden appearance of a highly diverse Middle Triassic coral fauna is that skeletonisation has been an ephemeral trait during the evolution of the Scleractinia [21]. Under this scenario, scleractinian lineages may have undergone skeleton loss in the face of global environmental instability [21], which would severely compromise fossil preservation. Consistent with this idea, some corals have been shown to undergo complete (but reversible) skeleton loss under acid conditions [22], whereas other species are apparently much less susceptible to skeleton dissolution [23].

**Figure 1:**
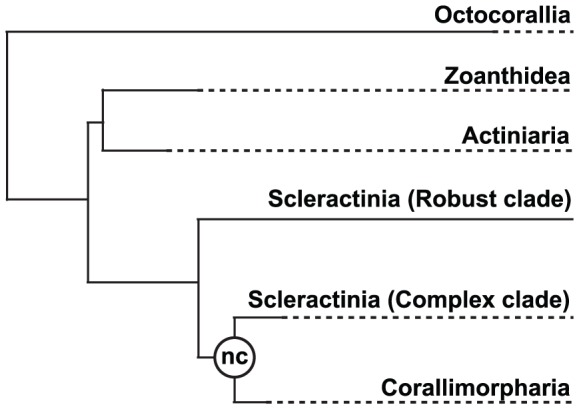
Phylogenetic relationships within the cnidarian Class Anthozoa according to the “naked corals” (nc) hypothesis (modified from [24]).

The “naked coral” hypothesis [24] is a topical extension of the idea of skeleton ephemerality in corals. Corallimorpharians, anthozoans that lack skeletons, have a close but unclear relationship to the Scleractinia. Corallimorpharians and scleractinians are very similar both in terms of anatomy and histology (see [92]), and these characteristics have in the past been used to argue for merging the orders [21], [26]. Medina et al. [24] conducted a phylogenetic analysis based on the proteins encoded by 17 complete mitochondrial (mt) genomes, which suggested that scleractinians are paraphyletic, corallimorpharians being more closely related to “Complex” than are “Robust” corals (Figure 1). The authors hypothesize that the Corallimorpharia (“naked corals”) may have arisen during the Cretaceous (110∼132 Mya) from a scleractinian ancestor that had undergone skeleton loss as a consequence of ocean acidification. A recent study [27] using complete mitochondrial genomes from a broad range of representative cnidarians also failed to unambiguously reject the “naked coral” hypothesis.

Here we applied a wide variety of analytical methods to a more comprehensive dataset of complete mitochondrial genome sequences (50 representative anthozoans) to better understand hexacorallian phylogeny. Whilst phylogenetic analyses based on amino acid (aa) data were for the most part consistent with the “naked corals” scenario (i.e. paraphyletic Scleractinia), it remains possible that the “naked corals” topology is an artefact caused by saturation, compositional biases or other violations of model assumptions. With the possible exception of cerianthiids [28], anthozoan cnidarians are thought to carry out mt DNA repair and thus differ from bilaterians *sensu stricto* in this respect. The main lines of evidence for repair are the extremely slow rate of evolution of the mt genomes of Anthozoa [29], [30] and the presence of a *MutS*-like gene in the octocoral mt genome [25], . We speculate that reduced efficiency of mt DNA repair in the “Robust” coral lineage could account for the observed anomalies in codon use and composition.

## Material and Methods

### DNA extraction and Polymerase Chain Reaction

Total genomic DNA was extracted using a Qiagen Qiamp or DNeasy Tissue Kit (QIAGEN). DNA concentrations were determined using a Nanodrop 1000 (Thermo Scientific) and an aliquot of each species total genomic DNA was diluted or concentrated to a final concentration of 40 ng/µl. Previously described primer sequences [32] were used to amplify the complete mt genomes of the following scleractinians: *Alveopora* sp.; *Astreopora explanata; A. myriophthalma; Isopora palifera*; and *I. togianensis*.

Two to three fragments (3∼9 kb) covering the entire mt genome of all but *G. hawaiiensis* were amplified by long Polymerase Chain Reaction (long-PCR) technique [33] from partial fragments of *rns*, *rnl* and *cox1* genes obtained from coral-specific primers and invertebrate universal primer [34], [35], [36]. Long-PCR were carried out using Takara La Taq using a slightly modified conditions from those recommended by the manufacturer as follows: 94°C for 1 min, then 30 cycles of 10 s at 98°C, 45 s at 62∼63°C, 14.25 min at 68°C for, and 10 min at 72°C. For *G. hawaiiensis* portions of *rnl, rns* and *cox1* were first amplified with the universal primers mentioned above, followed by the scleractinian universal primers CS-1 to CS-21 [37] that covered the entire mt genome. To obtain sequence from regions that did not yield product using these primers, nineteen specific primers were developed based on the sequences retrieved from *G. hawaiiensis* (Table S1). Polymerase Chain Reaction (PCR) were carried out using the Advantage2 polymerase kit (Clontech) under the conditions recommended by the manufacturer. PCR conditions were: 95°C for 5 min, then 30 cycles of 30 s at 94°C, 60 to 90 s at 54∼60°C, 90 s at 72°C, followed by 5 min at 72°C.

### Phylogenetic analysis

Resulting sequences were verified and assembled using Sequencher 4.8 (Gene Codes Corporation) and then analyzed in Vector NTI 9.0 (InforMax, Invitrogen life science software). Examination of open reading frames (ORFs) and codon usage, as well as other DNA statistics were performed using Dual Organelle Genome Annotator [38], Sequence Manipulation Suite v.2 [39], and MEGA5 [40]. In addition to the 6 new complete scleractinian mt genome sequences determined for this study, those of 25 other scleractinians, 12 corallimorpharians, 2 sea anemones, single antipatharian and zoanthid species, and 3 octocorals were obtained from public databases (Table 1).

**Table 1:** Mitochondrial genome sequence data included in the present analyses

Order	Species	size (bp)	GenBank accession #	Reference
Actiniaria				
	*Metridium senile*	17,443	NC000933	[78]
	*Nematostella* sp.	16,389	DQ643835	[24]
Alcyonacea				
	*Briareum asbestinum*	18,632	DQ640649	[24]
	*Pseudopterogorgia bipinnata*	18,733	DQ640646	[24]
	*Sarcophyton glaucum*	Incomplete	AF064823	[86]
Antipatharia				
	*Chrysopathes formosa*	18,398	NC008411	[87]
Corallimorpharia				
	*Actinodiscus nummiformis*	20,922		Lin et al. (submitted)
	*Amplexidiscus fenestrafer*	20,188		Lin et al. (submitted)
	*Corallimorphus profundus*	20,488		Lin et al. (submitted)
	*Corynactis californica*	20,632		Lin et al. (submitted)
	*Discosoma* sp.1	20,908	DQ643965	[24]
	*Discosoma* sp.2	20,912	DQ643966	[24]
	*Pseudocorynactis* sp.	21,239		Lin et al. (submitted)
	*Rhodactis indosinesis*	20,092		Lin et al. (submitted)
	*Rhodactis mussoides*	20,826		Lin et al. (submitted)
	*Rhodactis* sp.	20,093	DQ640647	[24]
	*Ricordea florida*	21,376	DQ640648	[24]
	*Ricordea yuma*	22,015		Lin et al. (submitted)
Scleractinia				
	*Acropora tenuis*	18,338	AF338425	[64]
	*Agaricia humilis*	18,735	DQ643831	[24]
	*Anacropora matthai*	17,888	AY903295	[32]
	*Alveopora* sp.	18,146		KJ634271
	*Astrangia* sp.	14,853*	DQ643832	[24]
	*Astreopora explanata*	18,106		KJ634269
	*Astreopora myriophthalma*	18,106		KJ634272
	*Colpophyllia natans*	16,906*	DQ643833	[24]
	*Euphyllia ancora*	18,875	JF825139	[37]
	*Fungiacyathus stephanus*	19,381	JF825138	[37]
	*Gardineria hawaiiensis*	19,429		Lin et al. (submitted)
	*Goniopora columna*	18,766	JF825141	[37]
	*Isopora palifera*	18,725		KJ634270
	*Isopora togianensis*	18,637		KJ634268
	*Madracis mirabilis*	16,951*	EU400212	[61]
	*Madrepora oculata*	15,839*		[65]
	*Montastraea annularis*	16,138*	AP008974	[62]
	*Montastraea faveolata*	16,138*	AP008978	[62]
	*Montastraea franksi*	16,137*	AP008976	[62]
	*Montipora cactus*	17,887*	AY903296	[32]
	*Mussa angulosa*	17,245*	DQ643834	[24]
	*Pavona clavus*	18,315	DQ643836	[24]
	*Pocillopora damicornis*	17,425*	EU400213	[61]
	*Pocillopora eydouxi*	17,422*	EF526303	[88]
	*Polycyathus* sp.	15,357*	JF825140	[37]
	*Porites okinawensis*	18,647	JF825142	[37]
	*Porites porites*	18,648	DQ643837	[24]
	*Seriatopora caliendrum*	17,010*	NC010245	[59]
	*Seriatopora hystrix*	17,059*	EF633600	[59]
	*Siderastrea radians*	19,387	DQ643838	[24]
	*Stylophora pistillata*	17,177*	EU400214	[61]
Zoanthidea				
	*Savalia savaglia*	20,764	NC008827	[89]

In order to make the analyses based on nucleotide and amino acid sequence data strictly comparable, the rRNAs, IGS, and tRNAs coding sequences were excluded from consideration. Therefore, for each species included in the present analysis, the data set included all protein-coding genes. The predicted amino acid sequences encoded by each of the 50 mt genomes were aligned using MAFFT v.5 [41]. These alignments were reverse translated to generate nucleotide sequence alignments, and phylogenetic inferences carried out on the concatenated amino acid and nucleotide alignments, removing all positions containing more than 50% gaps. The most appropriate model of nucleotide substitution was determined for the final alignment (totaling 11,802 bp) by the hierarchical likelihood ratio test implemented in MEGA5 as GTR+I+G (lnL -133020.1). Maximum Likelihood fits of 48 different amino acid substitution models using only positions that did not contain any gaps or missing data were calculated in MEGA5 [40]. There were a total of 3,666 positions (from the 3,934 aa) in this final dataset and JTT+G+I+F (lnL -51687.5) was chosen as the best evolutionary model.

Phylogenetic analyses were performed using PhyML [42] for Maximum Likelihood (ML) and MrBayes version 3.1.2 [43] for Bayesian Inference (BI). ML analyses were performed under the GTR model for nt alignments and JTT for aa alignments. For the BI, 2 runs of 4 chains were calculated for 10 million generations with topologies saved every 1,000 generations. One million generations were discarded as burn-in to ensure that the likelihood had plateaued and that the two runs had converged to less than 0.002 average standard deviation of split frequencies.

Given concerns for the influence of the long branch of the “Robust” scleractinian clade, ML phylogenetic analyses were repeated under several different scenarios for the nt data matrices as follow: i) different substitution model categories following Bayesian Information Criterion [BIC] and ML [lnL] recommendations; ii) systematically codon exclusion (1^st^, 2^nd^, and 3^rd^); iii) different nucleotide divergence rates across frames; iv) coding nucleotide data as purines and pyrimidies (RY-coding) (see [44], [45]) RY-coding excluding the 3^rd^ codon. Likewise, additional ML phylogenetic analyses of the aa final alignment included: i) coding aa using the common six groups that usually replace one another [46], [47], where MVIL were recoded as 1, FYW as 2, ASTGP as 3, DNEQ as 4, and RKH as 5, and C as 6; ii) to allow general-time-reversible (GTR) matrix to be used, the aa dataset was recoded to four categories instead of six. In this case, following [47] the aromatic (FYW) and hydrophobic (MVIL) amino acids were combined and the rare cysteine was considered as missing data. The four amino acid categories were named A, T, G, and C, respectively; iii) exclusion of all Phe, Ala, Thr and Tyr from the alignment, once the percentage of occurrence of these aa, especially of Phe and Ala, in the “Robust” scleractinian clade are significantly different once compared to all other hexacorallians included in the present analysis; iv) phylogenetic reconstructions using different evolutionary models as retrieved from results of the Bayesian Information Criterion [BIC] and ML [lnL] recommendations (JTT+G+I+F, cpREV+G+I+F, WAG+G+I+F, and Dayhoff+G+I+F); v) exclusion of Octocorallia sequences as outgroups; and vi) systematically exclusion of fast evolving sites. To find such sites, the mean (relative) evolutionary rate was estimated under the JTT+G+F in MEGA5, and a discrete Gamma (+G) distribution with 5 categories was used to model evolutionary rate differences among sites. Subsequently, 3 minimum evolution ML phylogenies were reconstructed systematically excluding all fast evolving sites that had means of >2.99, >1.99, and >1.49 respectively.

Trees with non-stationary, non-homogeneous models were computed using nhPhyML [48] with 5 categories of (G+C) content. Quartet puzzling with the Barry and Hartigan model was implemented using the PyCogent library [49], as was the Goldman [50] test.

Codon-based ML trees were inferred using CodonPhyml [51]. The results presented here used the Yap et al. model [52], but similar results were observed with other models. Codon-based trees were also inferred using MrBayes with a GTR substitution model and three categories of non-synonymous/synonymous ratios (M3 model).

For amino acids, phylogenies based on the CAT-GTR, CAT-Poisson and GTR models were inferred using PhyloBayes [53]. For each inference, the program was run until the effective size was greater than 300 and until the largest discrepancy across bipartition between runs was less than 0.1. Majority rule posterior consensus trees were built after deleting 1000 burn-in samples and taking every 10 generations.

Comparisons of topologies were carried out using the Approximately Unbiased, Kishino-Hasegawa and Shimodaira-Hasegawa tests implemented in the program Consel [54]. These tests compare the significance of the difference in likelihood of two competing topologies under the same model.

### Base Frequencies Distance Trees

The homogeneity of base frequencies among taxa is a major assumption of many molecular phylogenetic methods [55]. Therefore, changes in base composition between lineages can lead to errors in phylogenetic inference, particularly in the case of mt genome data (see [56]). In an attempt to assess the potential for compositional bias affecting the anthozoan phylogenetic inference, minimum evolution Base Frequencies distance trees (BF) were estimated using MEGA5 from matrices of pairwise BF distances.

Following Phillips et al. [45], BF distance was calculated for each taxon pair for each nucleotide category (i.e. *BF distance  =  ([{Ai − Aj} + {Ti − Tj} + {Ci − Cj} + {Gi − Gj}]/2*), where *i* and *j* are the frequencies of each corresponding nucleotide for the *i*th and *j*th taxa, respectively.

### tRNA and rRNA trees

For each of the 50 anthozoan species studied, the four mitochondrial genes encoding stable RNAs (i.e., 12S rRNA, 16S rRNA, *trnM*, and *trnW*) were retrieved, but the octocoral and actiniarian data were excluded from phylogenetic analyses because of the difficulty of generating unambiguous alignments when they were included. For these analyses, the scleractinian and corallimorpharian sequences were aligned and the antipatharian *Chrysopathes formosa* used as the outgroup. Each stable RNA sequence was aligned using essentially the same approach as for the protein-coding genes, the final alignments being 1,039 bp for 12S rDNA, 1,866 bp for 16S rDNA, 72 bp for *trnM*, and 70 bp for the *trnW*. Phylogenetic inferences were based on concatenated alignments and the most appropriate model of nucleotide substitution as determined by the hierarchical likelihood ratio test was GTR+G. ML (SH-like and 100 bootstrap) were performed using PhyML [42], and BI using MrBayes version 3.1.2 [43]. BI and ML analyses were performed using the GTR model as described above.

## Results and Discussion

Whereas previous analyses were based on a limited range of scleractinian and corallimorpharian mitochondrial genomes [24], [27], taxon sampling was increased in the present study to a total of 50 mt genomes, which included 12 corallimorpharians and 31 scleractinians (Table 1). Two taxa included in the present study are of particular evolutionary significance: *Corallimorphus profundus* and *Gardineria hawaiiensis*. Both anatomical [57] and molecular (this study) data suggest that *C. profundus* represents a deep-diverging corallimorpharian clade. *G. hawaiiensis* represents a lineage of scleractinians that is thought to have diverged prior to the “Complex”/”Robust” split [11], [17], [58].

### General characteristics of the mt genomes of hexacorallians

All of the hexacorallian mt genomes sequenced to date contain 13 protein-coding genes (*atp6* and *8, cox1-3, cob, nad1-6*, and *nad4L*), 2 genes encoding ribosomal RNAs (*rns* and *rnl*), and 2 encoding tRNAs (*trnW* and *trnM*), although members of the scleractinian genus *Seriatopora* have a duplication of *trnW* and thus have a total of 3 tRNA genes [59]. Whilst Hexacorallia in general display little variation in size of the mt genome, members of the Scleractinia are exceptional in having mt genomes ranging from >19.4 Kb in the “Basal” coral *Gardineria*, to <15 Kb in some “Robust” corals, those of “Complex” corals being intermediate in size (∼18.9–19.4 Kb) [37]. The size of each mt gene is also relatively stable across the range of hexacorallians, exceptions being *rnl* and *rns*, which vary by almost 500 and 700 bp respectively. As in Octocorallia [60], intergenic regions and introns (*cox1* and *nad5*) account for most of the observed variation in mt genome size. Some differences were apparent across hexacorallian orders, but gene organisation was remarkably uniform across the full range of Scleractinia, the only deviations from the canonical gene map [24], [32], [59], [61]–[64] being two azooxanthellate corals, *Lophelia pertusa* [90] and *Madrepora* spp. [65]. Likewise the majority (10 of 12) of corallimorpharian mt genomes conform to a distinct gene order, the exceptions being the azooxanthellate species *Corallimorphus profundus* and *Corynactis californica*.

**Figure 2:**
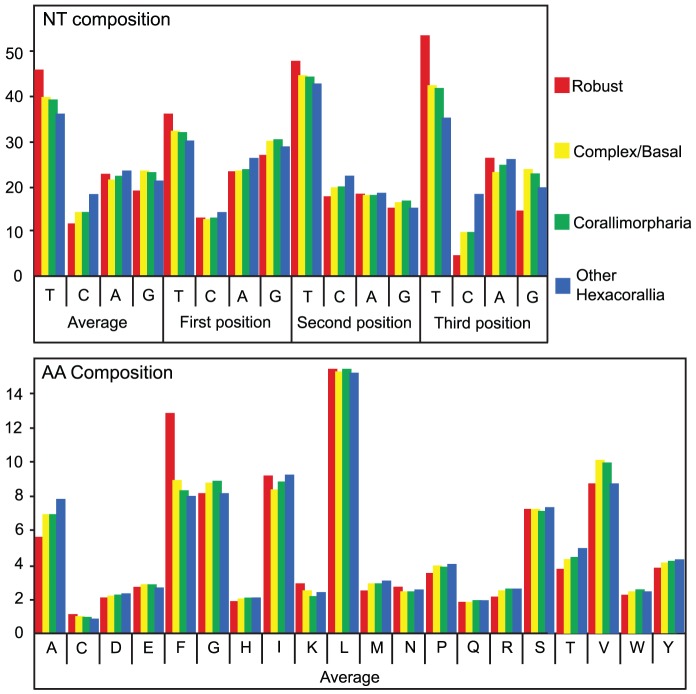
Nucleotide (upper) and amino acid (lower) content of the protein coding genes in the mitochondrial genomes of hexacorallians. The data shown are averages across the “Robust” corals (n = 14), basal and “Complex” corals (n = 17), corallimorpharians (n = 12) and other hexacorallians (n = 4).

The nucleotide composition of the mt protein-coding genes of hexacorallians has a clear (A+T)-bias, ranging from around 56% in the zoanthid *Savalia* to an average of 69% in “Robust” corals (Table 2 and Figure 2). The coding sequences of “Robust” corals have a high thymine and low cytosine content compared to other scleractinians (Figure 2). Surprisingly, this T-enrichment over other scleractinians is not restricted to silent codon positions, but is also clear at the first (5%) and second (3%) codon positions (Figure 2), resulting in over 400 aa substitutions in “Robust” corals relative to other hexacorallians (see also Table 2).

**Table 2:** Compositional biases of the mitochondrial genomes of the anthozoan Orders included in the present analysis.

			Nucleotide		Protein		
Sub-class	Order	Group	G+C (%)	A+T (%)	FYMINK (%)	GARP (%)	FYMINK/GARP
Octocorallia	Alcyonacea		35.33	64.66	30.54	21.96	1.39
Hexacorallia	Antipatharia		38	62	29.78	22.05	1.35
	Actiniaria		37.95	62.05	29.92	22.47	1.33
	Zoanthidea		43.9	56.1	29.4	23.09	1.27
	Corallimorpharia		37.95	62.05	28.73	22.15	1.29
	Scleractinia	Basal*	38.8	61.2	28.51	22.19	1.28
		Complex*	37.59	62.41	29.42	21.81	1.34
		Robust*	31.2	68.8	33.71	19.36	1.74

The figures shown are averages across the range of species included. For proteins, the comparisons are made between the (A+T)-rich amino acids FYMINK (Phe, Tyr, Met, Ile, Asn, and Lys), and the (G+C)-rich amino acids GARP (Gly, Ala, Arg, and Pro). Asterisks indicate groupings based on molecular data but whose taxonomic validity remains to be established.

### Contradictory phylogenies based on nucleotide or amino acid sequence data

**Figure 3:**
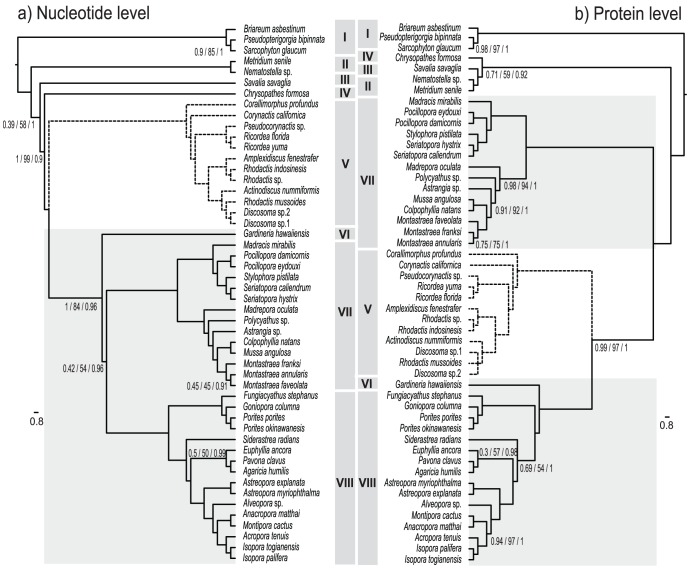
Phylogenetic analyses based on (a) the nucleotide sequences of the mitochondrial genes encoding proteins and (b) the amino acid sequences of the proteins encoded by the mitochondrial genomes. Values on the nodes indicate the non-parametric SH test and bootstrap replicates (ML), and posterior probability (BI) support respectively. Where no values are shown on a node, that edge was fully supported under all analyses. Dashed lines indicate the corallimorpharian clade. Light grey blocks identify the scleractinian clades. (I) Octocorallia used as outgroup; (II) Actiniaria; (III) Zoanthidea; (IV) Antipatharia; (V) Corallimorpharia; (VI) “Basal” Scleractinia; (VII) “Robust” Scleractinia; (VIII) “Complex” Scleractinia.

**Figure 4:**
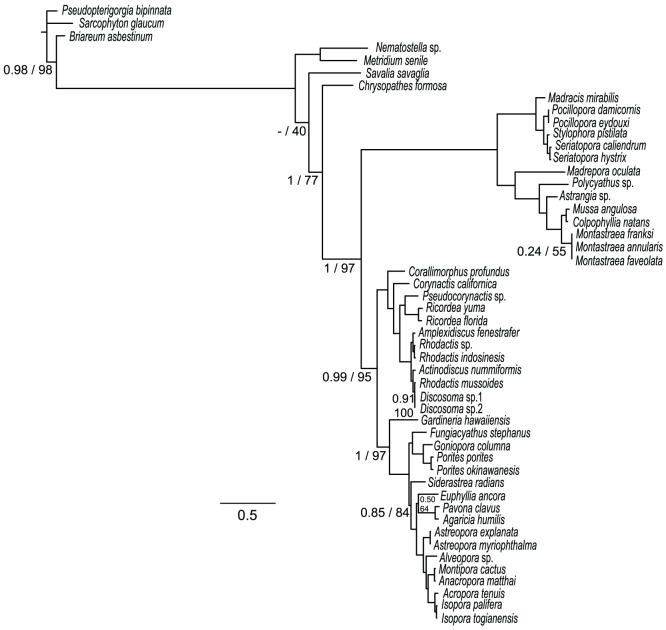
Codon-based phylogenetic analyses inferred using CodonPhyml – Yap et al. model [52] for Maximum Likelihood – and MrBayes – GTR substitution model and three categories of non-synonymous/synonymous ratios (M3 model). Values on the nodes indicate the non-parametric SH test and bootstrap replicates (ML), and posterior probability (BI) support respectively. Where no values are shown on a node, that edge was fully supported under all analyses.

Based on the final nt alignment (11,298 bp) consisting of all 13 protein-coding genes from 50 anthozoan mt genomes (Table 1), ML and BI recovered identical topologies and indicated that all anthozoan orders included in the analysis are monophyletic (Figure 3a). The topology shown was strongly supported, with only few nodes not having 100% support in both ML and BI. Scleractinians and corallimorpharians appear as sister groups. Whilst these results based on nt data flatly contradict the “naked coral” hypothesis [24], the application of the same phylogenetic methods to the corresponding amino acid sequence data (3,934 aa residues) consistently placed corallimorpharians as the sister group to the “Complex” Scleractinia, within the scleractinian clade (Figure 3b). In addition, the protein-based phylogenies differ in the positions of Actiniaria, Zoanthidea, and Antipatharia, and also in placing *G. hawaiiensis* as a member of the “Complex” corals instead of forming a basal scleractinian lineage [11], [17]. Codon-based phylogenies also strongly support the grouping of corallimorpharians with “Complex” corals (Figure 4). The significance of the difference in likelihood between to the competing topologies is shown on table (Table 3). The preference for the “naked coral” topology is highly significant for the trees based on amino acids and codons, whereas the significance of the difference is weaker for nucleotide-based trees.

**Table 3:** Comparison of the two competing topologies (scleractinian monophyly, SM, and “naked coral”, NC) using the Approximately Unbiased (AU), Kishino-Hasegawa (KH) and Shimodaira-Hasegawa (SH) tests for a variety of tree reconstruction methods.

	Best ML topology	AU	KH	SH
Nucleotides (GTR+G+I+F)	SM	0.10	0.11	0.11
Nucleotides (GG98)	NC	0.33	0.32	0.32
Codons (YAP+W+K+F)	NC	0.03	0.03	0.03
Amino Acids (JTT+G+I+F)	NC	0.007	0.008	0.008
Nucleotides (rRNA, tRNA, GTR+G+I+F)	SM	0.009	0.013	0.013
Nucleotides (rRNA, tRNA, nhPhyML)	SM	0.086	0.094	0.094

The p-values denote the probability that the best ML topology is equivalent to the alternative topologies. Unless otherwise indicated, the trees were based on the alignment of protein coding genes.

To better understand the basis of these contrasting results, we examined the potential for artifacts to arise as a result of the analytical methods or biases in the datasets.

### Use of different substitution models and removal of rapidly evolving sites

In the case of both nt and aa analyses, changing the outgroup had no effect on topology, and neither did the use of different substitution model categories. For nucleotides, the models validated included: (i) parametric GTR [93] with gamma distribution of rates among sites; (ii) TN93 [66] with gamma distribution and invariable sites; (iii) and HKY [67]. Using amino acid data, the JTT+G+I+F [68], cpREV+G+I+F [91], WAG+G+I+F [69], and Dayhoff+G+I+F [70]. Furthermore, in order to verify differences in evolutionary models selection, the same analyses were extended with the exclusion of all sequences from “Robust” corals from the dataset. However, these new analyses recovered similar results as described above.

Next, standard rate effects were examined. Potential saturation effects in the nt data were examined by systematically excluding the 1^st^, 2^nd^, and 3^rd^ codon positions from analyses, but the ML topology retrieved and statistical support for nodes did not differ significantly from those shown in Figure 3a. In fact, exclusion of the 3^rd^ codon position improved support for some nodes (Figure S1). Removing the most rapidly evolving sites in the aa alignment [47] also had no effect on the topology. This approach involved estimation of the mean (relative) evolutionary rate (ER) for each site under the JTT+G+F [68] model and then excluding those sites with ERs of ≥2.99, ≥1.99, or ≥1.49 (note that sites displaying ER >1 are evolving faster). The percentages of sites excluded in these cases were 18.3%, 23.1%, and 32.4% respectively; 1,275 of the 3,934 sites could therefore be excluded from the analyses without influencing the overall topology (Figure S2).

### Compositional bias effects: nucleotides

Having investigated potential artifacts arising from standard rate effects, the effects of compositional heterogeneity in the nucleotide and amino acid data were examined. In the case of the nt alignment, this involved RY coding [44], [45], with or without exclusion of the third codon position from the resulting alignment (Table S2), which also resulted in scleractinian monophyly (Figure S3).

As noted above, the mt genomes of “Robust” corals do differ significantly from those of all other hexacorallians in terms of nucleotide composition, and this has consequences for both codon use and amino acid composition in the proteins that it encodes. Figure 2, Table 2 and Figure S4 show the overall base composition of mt protein-coding genes of the anthozoans included in the present analysis, and also the percentage of each base occurring in the 1^st^, 2^nd^, and 3^rd^ codon positions.

Whereas most hexacorallians have (A+T) contents around 62% (hence (A+T)/(G+C) of around 1.63), “Robust” corals have a significantly higher (A+T content ((A+T)/(G+C) = 2.20). Consequently, the (A+T)-skew is >6% higher in “Robust” corals than in all other hexacorallians (Figure S4). This bias is asymmetrically distributed, such that in “Robust” corals the coding strand is anomalously high in thymine and low in cytosine. Such heterogeneities in base composition are a potential source of error in phylogenetic analyses [71].

In order to take into account this variability in nucleotide composition, we used the GG98 non-stationary, non-homogeneous model [72] implemented in the nhPhyML software [48]. In this approach the “naked coral” topology has the highest likelihood, but the difference in likelihood of the two competing topologies is not statistically significant (Table 3).

We further explored the effect of compositional heterogeneity using the Barry and Hartigan model [4] implemented in the PyCogent library [49]. The Barry and Hartigan model is the most general (makes the fewest assumptions) substitution model for nucleotides. It allows for non-reversible and non-stationary processes on every branch of a phylogeny and does not assume the process is time-homogeneous within or between branches. The complexity of this model precluded tree inference; instead, 1,000 quartets, each comprising a “Robust” coral, a “Complex” coral, a corallimorpharian and an outgroup, were sampled. The majority (94%) of these quartets grouped complex corals with corallimorphs and “Robust” corals with the outgroup.

**Figure 5:**
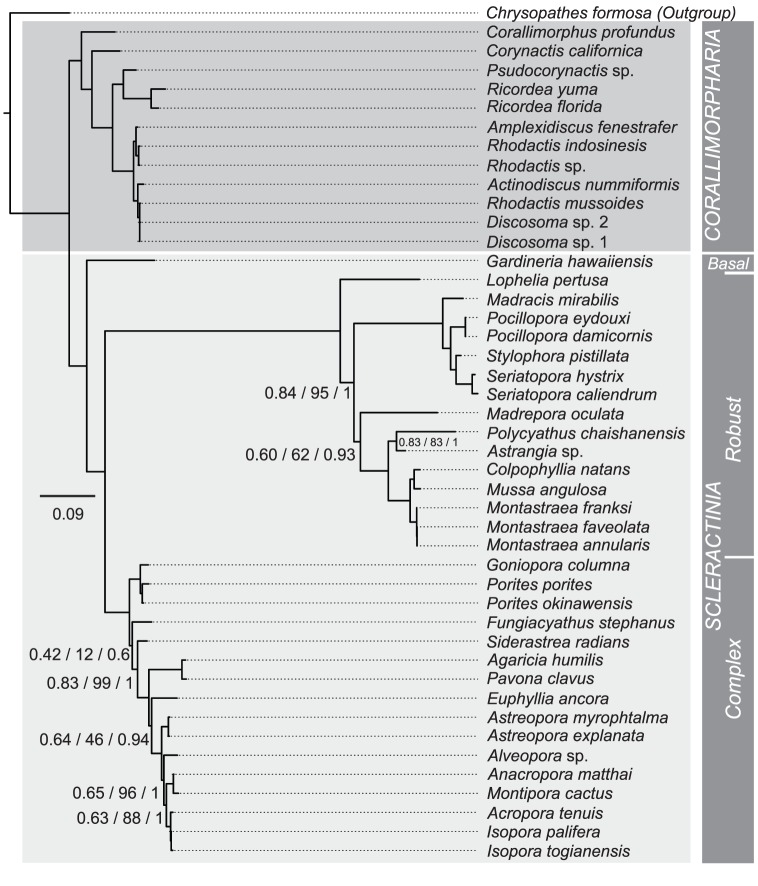
Phylogenetic analyses of the mitochondrial sequences encoding stable RNAs (12S rRNA, 16S rRNA, *trnM*, and *trnW*). Values on the nodes indicate the non-parametric SH test and bootstrap replicates (ML), and posterior probability (BI) support respectively. Where no values are shown on a node, that edge was fully supported under all analyses. Large boxes indicate the Corallimorpharia (dark-gray) and Scleractinia (light-gray) clades. Note that deep-water azooxanthellate species (*Corallimorphus profundus* and *Gardineria hawaiiensis*) represent the earliest diverging branches for Corallimorpharia and Scleractinia respectively.

Taken together, these results based on models that do not assume compositional homogeneity or time reversibility suggest that the strong support of nt-based phylogenies for scleractinian monophyly might be an artifact of sequence composition. However, phylogenetic analyses carried out on a concatenated rRNA and tRNA alignment recovered a monophyletic Scleractinia clade with high statistical support irrespective of the method of analysis (Figure 5 and Table 3). Using this alignment, quartet puzzling with a Barry and Hartigan model also favored scleractinian monophyly in 99.9% of cases.

### Compositional bias effects: codons and amino acids

**Figure 6:**
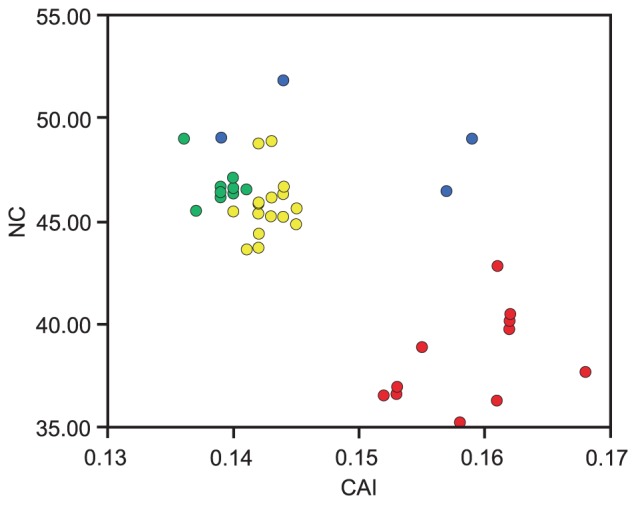
Codon usage in hexacorallian anthozoans. CodonW [73] was used to estimate codon usage biases; default settings were used in calculation of the codon adaptation index. NC: effective codon number. CAI: codon adaptation index. Colour coding and taxon choice is as shown in Figure 2.

Clear biases in codon usage are seen throughout the Hexacorallia, but in “Robust” corals, this bias is more extreme, as evidenced by consistently lower effective number of codon (NC) scores and higher codon adaptation indices (calculated using CodonW [73]) than other scleractinians or corallimorpharians (Figure 6). For some amino acids, codon usage in “Robust” corals differed markedly from that in the other hexacorallians for which data are available (Figure 2). This pattern was also seen in the AT skew analyses (Figure S4).

In both aa and codon based phylogenies (Figures 3b and 4 respectively), the branch leading to the “Robust” coral clade is disproportionately long due to the presence of a large number of aa substitutions that are shared across most or all members of this clade but are not seen in other hexacorallians. Phenylalanine is the second most abundant aa in the mt-encoded proteins of “Robust” corals, and is approximately 1.5-fold more abundant in members of this clade, compared to other hexacorallians (Figure 2). The observed increase in abundance of (TTT-encoded) Phe residues in “Robust” corals suggests that shifts in nt abundance may have forced the large number (>18%) of changes at the aa level that are unique to and shared within the “Robust” clade. (A+T)-rich amino acids (FYMICK) are more abundant, and (G+C)-rich amino acids (GARP) less abundant in the proteins encoded by the mt genomes of “Robust” corals than in other hexacorallians (Table 2).

To further investigate the possibility of long-branch artifacts on the amino-acid-based phylogenies, the PhyloBayes program was employed to infer trees using the CAT-Poisson, CAT-GTR and GTR models [74]. The CAT model allows different positions to evolve using a distinct substitution process and to have a different equilibrium frequency. It has been shown that this type of model is less sensitive to saturation and can reduce long-branch artifacts [75]. Table 4 summarizes the topologies and posterior probabilities obtained with this approach. The CAT-Poisson and CAT-GTR models tend to support scleractinian monophyly, while the GTR model is consistent with the naked coral hypothesis. These results are consistent with a recent study [27], where the monophyly of scleractinians received a modest support from the CAT-GTR model, but was not supported by the GTR model or by any nucleotide-based phylogenies.

**Table 4:** Posterior probability of the topologies recovered by different models using Phylobayes (SM: scleractinian monophyly, NC: naked coral).

Model	Topology	Posterior probability
CAT GTR	SM	0.56
CAT Poisson	SM	0.94
GTR	NC	1

### Hypothesis: did impaired mt DNA repair and constraints on tRNA uptake result in the altered amino acid usage in “Robust” corals?

If mt DNA repair is an ancestral property within Anthozoa [76], then the faster rate of mt genome evolution and differences in base composition that characterize “Robust” corals may reflect decreased efficiency of the repair process in this clade (see also [77]); if the resulting mismatches were not repaired, spontaneous deamination of cytosine to uracil could account for the atypical base composition. A consequence of the atypical base composition in the mt genomes of the “Robust” corals (higher T and lower C when compared to other hexacorallians) is a shift in the amino acid composition of mt proteins towards those encoded by T-rich codons.

The mt genomes of Anthozoa differ from those of the Bilateria in encoding only two tRNAs – tRNAMet and tRNATrp [78], hence in anthozoans most of the tRNAs required for mt translation must be imported. The mt tRNA uptake systems of anthozoans clearly have specificity, as codon use differs between mitochondrial and nuclear genes in *Acropora* despite similar overall base composition; for example, TTT being the strongly favoured Phe codon in mt-genes but bias being much less apparent in the case of nuclear genes [79], [80].

“Robust” corals consistently display higher %(A+T) (around 5 to 6% higher than in “Complex” corals, for example) than either corallimorpharians or “Complex” corals, the most obvious difference being an increased frequency of thymine at third codon positions on the coding strand. In organisms that must import most tRNAs into mitochondria, changes in the base composition of the mt genome may lead to changes at the amino acid level in the proteins that they encode; the higher % (A+T) in the mt genomes of “Robust” corals not only drives protein coding sequences towards (A+T)-rich codons but may also force non-silent changes towards amino acid residues that are encoded by (A+T)-rich codons [81]. Such a mechanism could account for the higher abundance of phenylalanine residues in proteins encoded by the mt genomes of “Robust” corals, due to the increased frequency of TTT codons. Hence many of the amino acid substitutions unique to the proteins encoded by the mt genomes of “Robust” corals likely reflect the compound effects of base composition changes and the constraints under which tRNA uptake operates. We hypothesize that these amino acid substitutions bias phylogenetic analyses based on mitochondrial amino acid sequence data, obscuring relationships amongst the major scleractinian clades and corallimorpharians.

Consistent with compositional biases affecting analyses based on amino acid sequence data, phylogenetic analyses based on the mitochondrial rRNA and tRNA sequences consistently resulted in monophyletic Scleractinia (Figure 5). Furthermore, BF distance topologies inferred to assess the potential for compositional bias to affect phylogenetic inference suggested that overall, the aa data slightly favors the “naked coral” hypothesis, whereas nt based BF topology appears to be more homogeneous (Figure S5). Although the issue remains equivocal, molecular support for the “naked coral” hypothesis may therefore be an artifact resulting from compositional bias and saturation between the two major scleractinian clades. Note that these results do not challenge the issue of skeleton ephemerality sensu Stanley and Fautin [21] in Scleractinia, but imply that corallimorpharians are not descendants of a scleractinian that had undergone skeletal loss.

Changes in DNA repair mechanisms in some clades would result, in an evolutionary history, in violation of the assumptions of the models used for inference. With the exception of that of Barry and Hartigan, all models of substitution assume time-homogeneity both within and between branches [82]. All models of recoded sequences, including the aa substitution models, are non-Markovian, which results in a non-linear relationship between the true substitution dynamics operating on the nucleotide sequences and what is inferred using these models [83]. Thus, the ambiguous results outlined above could be a consequence of a poor fit between the models (despite these being selected as fitting the data best from the collection of models tested) and the evolutionary process. We evaluated how well the models fit compared to the best-possible likelihood, as proposed by Goldman [50] and implemented in the PyCogent library [49]. In brief, this test compares the difference in likelihood between the fitted model and the best-possible likelihood (calculated without assuming any phylogenetic relationship between the sequences) to the distribution of difference between these two likelihoods that one would expect if the data were generated according the fitted model (Figure S6). These tests can only be carried out in the maximum likelihood framework, thus for nt data the GTR and Barry-Hartigan models were tested and for aa data the JTT model. P-values were computed based on 200 Monte Carlo simulations, and for all models the fitted likelihoods were vastly inferior compared to the best possible, confirming a poor agreement between the data and the models used, even the most general one (with the fewest assumptions).

## Conclusion

The hypothesis outlined here – that, for hexacorallians, analyses based on mitochondrial sequences may be intrinsically biased - can and should be tested when appropriate nuclear sequence data are available for a wide range of corals and corallimorpharians. Molecular phylogenetics has led to radical revisions in thinking about coral evolution, but such analyses have largely been based on mt sequence data. Similar problems with mitochondrial sequences have been highlighted for a number of other animal groups including mammals [84] and beetles [85]. Given the above concerns, it is important that the bias towards mt data is redressed, and coral phylogenetics more broadly be based on a wide range of nuclear loci.

## Supporting Information

Figure S1 **Phylogenetic analyses based on the nucleotide sequences of the mitochondrial genes encoding proteins with the exclusion of the 3^rd^ codon position.** Values on the nodes indicate the posterior probability (BI) support. Where no values are shown on a node, that edge was fully supported under all analyses.
(EPS)

Figure S2 **Phylogenetic analyses based on amino acid sequences of the mitochondrial genes encoding proteins removing the most rapidly evolving sites based on the mean (relative) evolutionary rate (ER) for each site under the JTT+G+F model.** A, B, and C indicate the topologies recovered excluding all those sites with ERs of ≥2.99, ≥1.99, or ≥1.49 respectively. For each ER reconstruction the topology with the highest log likelihood is shown. Values on the nodes indicate the ML bootstrap (100 replicates) support.
(EPS)

Figure S3 **Phylogenetic analyses based on the nucleotide sequences of the mitochondrial genes encoding proteins re-coded as purines and pyrimidies (RY-coding see** [44], [45] **) with the exclusion of the third codon position from the resulting alignment.** Values on the nodes indicate the posterior probability (BI) support. Where no values are shown on a node, that edge was fully supported under all analyses.
(EPS)

Figure S4 **Graphical representation of (G+C)- (red line) and (A+T)- (blue line) skew calculated on the whole mitochondrial genome of all species included in the present analysis.** The (A+T)-skew is >6% higher in “Robust” corals than in all other hexacorallians included in the present analyses (highlighted in yellow).
(EPS)

Figure S5 **Minimum evolution tree on BF distances from the complete mt protein coding DNA sequences.** Topologies are based on nucleotide BF distances (left topology) and aa BF distances (right topology). AA compositional bias slightly favors the “naked coral” hypothesis (yellow box) whereas nt based BF topology appears to be more homogeneous.
(EPS)

Figure S6 **Empirical distribution of the difference between the likelihood of the fitted model and the best possible likelihood (the product of column pattern frequencies).** In each case the arrow indicates the observed value of that difference. The distributions are shown for the nucleotide alignment of the protein coding sequences with the GTR model (A) and the Barry and Hartigan model (B), for the amino acid alignment with the JTT model (C), and for the RNA alignment with the GTR model (D).
(EPS)

Table S1 **Primer names and sequences used for the amplification/sequence of the mitochondrial genome of** ***Gardineria hawaiiensis*** **.** The position and amplicons length of primers designed in the present study or the reference for previously published primers are provided.
(DOC)

Table S2 **Alignment of the nucleotide sequences from the mitochondrial genes encoding proteins re-coded as purines and pyrimidines (RY-coding see** [44], [45] **).**
(TXT)
