# Supplementary material for: The “Naked Coral” Hypothesis Revisited – Evidence for and Against Scleractinian Monophyly
Source: PLoS One. 2014 Apr 16;9(4):e94774. doi: 10.1371/journal.pone.0094774 (PMC3989238; doi:10.1371/journal.pone.0094774)
Supplement: Table S1 — Primer names and sequences used for the amplification/sequence of the mitochondrial genome of Gardineria hawaiiensis . The position and amplicons length of primers designed in the present study or the reference for previously published primers are provided. (DOC) [file pone.0094774.s007.doc]

Supplementary table S1 – Primer names and sequences used for the amplification/sequence of the mitochondrial genome of *Gardineria hawaiiensis*. The position and amplicons length of primers designed in the present study or the reference for previously published primers are provided.

| Primer | Sequence (5’ to 3’) | Position (bp) or  Reference | Length (bp) |
| --- | --- | --- | --- |
| MVK-1F | GCTGGTGGATCTACCGTCTC | 2102-2121 | 749 |
| MVK-1R | GAATGTGGACTGGGGTCACT | 2837-2851 |  |
| MVK-2F | TTATGTCCGCTGAGCCTTTT | 5148-5167 | 1150 |
| MVK-2R | TTTTCACCATCCTCTCTCGG | 6279-6298 |  |
| MVK-3F | CCGAGAGAGGATGGTGAAAA | 6279-6298 | 784 |
| MVK-3R | AGCCCCCATAAACAACAGC | 7045-7063 |  |
| MVK-4F | GTGGGGGAACTGGCTCTT | 8792-8809 | 900 |
| MVK-4R | TCAAACCACACCCAACTCAA | 9673-9692 |  |
| MVK-5F | TTGAGTTGGGTGTGGTTTGA | 9673-9692 | 2073 |
| MVK-5R | CGCCTATTCTCACTCCAACC | 11728-11746 |  |
| MVK-5bF | GGCGACTGGGTTCCATGGTT | 9941-9960 | 1200 |
| MVK-5bR | CTATTAAGTGCCACCCCTCAGCATCC | 11116-11141 |  |
| MVK-6F | ATGCCCCTATTTGTGTGGTC | 13963-13982 | 2115 |
| MVK-6R | AAAGGAGGGGAAAGATGGAC | 16059-16078 |  |
| MVK-6bF | GCCCATTGGTTGATCGGGTTT | 14561-14582 | 750 |
| MVK-6bR | TGCTTCTCAAGAACCAACGCCAATAC | 15286-15311 |  |
| MVK-7F | AAGTGTGTTAGGCAGGGGAAT | 17389-17408 | * |
| MVK-8F | CTCTTTGGGGTGGTCTTTGG | 18695-18714 | 664 |
| MVK-8R | CCGTCTGCTTTATTCCTCTACA | 19338-19359 |  |
| LCO1490 | GGTCAACAAATCATAAAGATATTGG | Folmer et al. (1994) | 710 |
| HCO2198 | TAAACTTCAGGGTGACCAAAAAATCA | Folmer et al. (1994) |  |
| ANTMT12SF | AGCCACACTTTCACTGAAACAAGG | Chen & Yu (2002) | 920 |
| ANTMT12SR | GTTCCCYYWCYCTYACYATGTTACGAC | Chen & Yu (2002) |  |
| LP16SR | TCCCCAGGGTAACTTTTATC | Le Goff-Vitry et al. (2004) | * |
| CS-F1 | AAGCCATGTTAGTTAATCGAGTG | Lin et al. (2011) | 984 |
| CS-R1 | GATCAACCCAATCGAAACTTCA | Lin et al. (2011) |  |
| CS-F2 | CCATTGCTTATCACAGTAGCT | Lin et al. (2011) | 1175 |
| CS-R2 | TAATGCATGGACAAAAAGCACC | Lin et al. (2011) |  |
| CS-F3 | CATGTAGAGGGGTCAAATAGTCC | Lin et al. (2011) | 1266 |
| CS-R3 | CCAGATGAAAGTGCACCTAA | Lin et al. (2011) |  |
| CS-F4 | GTGGCATTAGGAAGTCTTTGT | Lin et al. (2011) | 1031 |
| CS-R4 | ATGGGCTAATTGCAACCATA | Lin et al. (2011) |  |
| CS-F5 | TTTGGGTAAGTGGTTGGTT | Lin et al. (2011) | 1019 |
| CS-R5 | GAATTAGTCAAGGCGATCAGA | Lin et al. (2011) |  |
| CS-F6 | TATGATCATCTTCATGGTGTCG | Lin et al. (2011) | 1220 |
| CS-R6 | GGGATCAATATGCCCTCAAA | Lin et al. (2011) |  |
| CS-F7 | GGCTTTTGATTTAGAGGGACA | Lin et al. (2011) | 1123 |
| CS-R7 | CTGCCCCAAAACTAATTCGA | Lin et al. (2011) |  |
| CS-F8 | TTATGTTGGGTGTTGTAGCTG | Lin et al. (2011) | 994 |
| *Supplementary table S2 - Continued* | | | |
| CS-R8 | TCTGCTGGCACTTAATTTGACG | Lin et al. (2011) |  |
| CS-F9 | GGACCACCTTGCTTATGATG | Lin et al. (2011) | 1164 |
| CS-R9 | GGATGACATAAAACAGTTCGCA | Lin et al. (2011) |  |
| CS-F10 | GTCGTAACATAGTGAGGGTGA | Lin et al. (2011) | 1110 |
| CS-R10 | TCTTGCAAACCCAAGTGTCA | Lin et al. (2011) |  |
| CS-F11 | CGAGTTGGTATTGGCATTTTG | Lin et al. (2011) | 1098 |
| CS-R11 | CGCAACCATAATAGCTAAACCA | Lin et al. (2011) |  |
| CS-F12 | CCAGGGACGTTTTATGGTCA | Lin et al. (2011) | 1228 |
| CS-R12 | GACCCCGCACTTAAGAACAATA | Lin et al. (2011) |  |
| CS-F13 | GGTAAAACAACCGGATCGAG | Lin et al. (2011) | 968 |
| CS-R13 | ACTAAATTCCAAGAACCCTCATG | Lin et al. (2011) |  |
| CS-F14 | ATGGGGTTTCCTTATTTAACAGG | Lin et al. (2011) | 726 |
| CS-R14 | TTGAAGGCTAACGGTCTACT | Lin et al. (2011) |  |
| CS-F15 | CATGAGGGTTCTTGGAATTTAGT | Lin et al. (2011) | 1036 |
| CS-R15 | TAACATACTGAAGGCTGTACCG | Lin et al. (2011) |  |
| CS-F16 | TTAGGTTAAAGTAGACCGTTAGCC | Lin et al. (2011) | 1018 |
| CS-R16 | ATCCGTTAAAAGCATGGTTATGG | Lin et al. (2011) |  |
| CS-F17 | GGATGAACGGTTTATCCTCCT | Lin et al. (2011) | 1508 |
| CS-R17 | GCAGTAAAATATGCTCTTGTGTCC | Lin et al. (2011) |  |
| CS-F18 | GGACACAAGAGCATATTTTACTG | Lin et al. (2011) | 976 |
| CS-R18 | CTACTTACGGAATCTCGTTTGA | Lin et al. (2011) |  |
| CS-F19 | GTGAGTCATCGGGCTCATG | Lin et al. (2011) | 1065 |
| CS-R19 | ACAGTCTGTTCTACTACCAAGC | Lin et al. (2011) |  |
| CS-F20 | GCTTGGTAGTAGAACAGACTGT | Lin et al. (2011) | 1013 |
| CS-R20 | AACATCGAGGTCGCAAACAT | Lin et al. (2011) |  |
| CS-F21 | AAAGCGTGGTAACACAGCTT | Lin et al. (2011) | 1062 |
| CS-R21 | CAACTGTGCAGACTTTCCAA | Lin et al. (2011) |  |
